# Supplementary material for: Prevalence of Leishmania RNA virus in Leishmania parasites in patients with tegumentary leishmaniasis: A systematic review and meta-analysis
Source: PLoS Negl Trop Dis. 2022 Jun 8;16(6):e0010427. doi: 10.1371/journal.pntd.0010427 (PMC9236231; doi:10.1371/journal.pntd.0010427)
Supplement: Attachment — Submitted filename: point-by point response.docx [file pntd.0010427.s003.docx]

# **Response to the Editor:**

We thank the editor for the important comments to improve our manuscript and we address them as follows: The changes and corrections are highlighted in yellow in the revised manuscript.

**Editor’s comment:** The review cannot be based only on cutaneous leishmaniasis, since there are data associating mucosal leishmaniasis and the presence of *LRV* and, therefore, the word cutaneous should be replaced by tegumentary in the title of the manuscript

**Response:** In response to the editor’s comment we have now changed the title of our study in the revised version of the manuscript to indicate which forms of cutaneous leishmaniasis were analyzed in our study. We replaced the word cutaneous by tegumentary in the title. The amended title of the revised manuscript is *“Prevalence of Leishmania RNA virus in Leishmania parasites in patients with tegumentary leishmaniasis: a systematic review and meta-analysis”.*

We have also defined the term “CL” in the revised manuscript to clarify that this comprises the different clinical manifestations of cutaneous leishmaniasis.

**Response to reviewers’ comments**

We thank all the reviewers for their helpful comments to improve our manuscript and we address them as follows:

**Reviewer 1:**

**Abstract**

**Reviewer’s comment**: total of 1159 samples from 24 studies were tested’ makes it sound as if you tested them.

**Response**: We have changed the sentence from: “A total of 1159 samples from 24 studies were tested” to: “A total of 1215 samples from 24 studies were included” (Incorporated in page 2, line 39)

**Author summary**

**Reviewer’s comment**: ‘It needs further studies to well understand’ would read better as ‘It needs further studies to better understand…’

**Response:** Following the reviewer’s suggestion we have changed the sentence to: “Further studies are needed to better understand the role of the virus in the clinical presentation of CL*...* (Incorporated in page 4, lines 69 and 70 the revised manuscript).

**Introduction**

**Reviewer’s** **comment:** Your opening sentence is too vague …

**Response:** In response to the reviewer’s comment we have now changed the opening sentence of the introduction in the revised manuscript as follows: The different clinical manifestations of tegumentary leishmaniasis comprise localized (CL) mucocutaneous (MCL) and diffuse cutaneous leishmaniasis (DCL); they are neglected tropical diseases affecting millions of people worldwide. *Leishmania* species probably co-evolved with mammals [1] and have been identified in humans from at least 4 millennia before present [2]. (page 5, lines 75-79).

**Reviewer’s** **comment:** multiplenon-ulcerative nodular lesionswith.  should be: multiple non-ulcerative nodular lesions with lar

**Response:** Thank you for pointing out the mistakes. We have now corrected the typing errors in the revised manuscript (page 6, lines 109 and 110)

**Methods**

**Reviewer’s** **comment**: Can you be clearer about your search strategy.

**Response**: As requested, we have now clarified in more detail our search strategy in the revised version of our manuscript (page 8, lines 143 and 144).

**Reviewer’s** **comment:** ‘The articles were independently reviewed by two investigators (EY, EN)’.  What did you do if you disagreed?

**Response:** As requested, we have now explained in the revised manuscript how disagreements were resolved. “Disagreements between the two investigators were resolved by discussions with all authors of this study until consensus was reached” (page 9, lines 165 and 166).

**Discussion**

**Reviewer’s** **comment**: ‘The detection of the *LRV* from the lesion biopsies would be much easier and help to run more samples in short period of time in areas with poor laboratory setup.’  I don’t understand this sentence. Please clarify.

**Response:** We have now clarified it in the revised manuscript as follows: The detection of the *LRV* directly from the lesion biopsies would be much easier than detecting it from cultured parasites since it does not require parasite culturing and would help to run more samples in a short period of time in areas with poor laboratory setup (page 21, lines 352-355).

**Reviewer 2**

**Introduction**

**Reviewer’s** **comment:** line 5 not necessarily leishmaniasis is prevalent in the poorest communities

**Response:** In response to the reviewer’s comment we have changed it to:” it impacts mostly on the poorest community. (page 5, line 81).

**Reviewer’s** **comment:** paragraph 2 line 5 there is no consensus that *LRV* can define the clinical form.

**Response:** We have now pointed out in the revised version that here is no consensus that *LRV* can define the clinical form: “However, there is no consensus on a defining role of *LRV* in disease progression, severity, metastasis, and treatment failure. Valencia *et al*., 2022 did not find clinical, parasitological or immunological evidence supporting the hypothesis that *LRV1* is a significant determinant in the pathobiology of American Tegumentary Leishmaniasis.” incorporated in in the revised manuscript (page 5, lines 97 and 98, page 6, lines 99-1011.

**Reviewer’s** **comment:** Paragraph 4 line 8 the authors could clarify whether the isolates were obtained from patients.

**Response:** As requested, this has now been clarified in the revised manuscript (page 7, line 132).

**Limitation of analysis**

**Reviewer’s** **comment:** There are few published studies available to be analyzed

**Response:** This has now been clearly stated in the revised version in the limitation section. We also included a recently published article, Valencia *et al*., 2022 (page 15, Table 1, last column, page 25 reference no 15). As indicated in the inclusion and exclusion criteria we did not include case reports, experimental studies because they cannot meet our aim.

**Methods**

**Reviewer’s** **comment**: 2.1 it in the search strategy, it was not clear whether the keywords were used separately, together or concatenated.

**Response**: We have now clarified in more detail our search strategy in the revised version of our manuscript (page 8, lines 143 and 144). Please see also our response to reviewer 1 (can you be clearer about your search strategy)

**Reviewer’s** **comment**: 2.2 why? all in vitro and animal model studies were eliminated, as these studies can be done with patient isolates

**Response:** In vitro studies and experimental work in animal models were excluded because the aim of this systematic review and meta-analysis is to determine the prevalence of *Leishmania RNA Virus* in *Leishmania* parasites isolated from patients.

**Reviewer’s** **comment 2.2**? Paragraph 2 lines 6 and 7 authors could provide the exclusion criteria made at this stage of the analysis

**Response:** As requested, the exclusion criteria is indicated in the revised manuscript (page 9, lines 162-164)

**Reviewer’s** **comment:** 2.3 line 1 authors could provide the data extraction form and criteria.

**Response:** As requested**,** the data extraction form is attached as supporting information.

**Reviewer’s** **comment:** line 9 authors could make it clear if the "checklist-JBI" were used as exclusion criteria.

**Response:** Yes**,** the checklist-JBI was used to appraise the quality of articles. Articles with low quality were excluded, this is incorporated in the revised manuscript (page 9, lines 177 and 178).

**Results**

**Reviewer’s** **comment:** 3.1. line 4: the authors could indicate that these results are described in figure 1.

**Response:** As requested, we added this information (Fig1) to line 202 in the revised manuscript.

**Reviewer’s** **comment** 3.1: line 5 the authors can describe how the 83 studies from the last 107 were excluded and indicate that the exclusion criteria is described in fig 1.

**Response:** As requested, we have now added to the text how the 83 studies were excluded from the 107 (lines 204-206) and that the exclusion criteria are detailed in Fig 1 in the revised manuscript (page 11, lines 204-207)

**Reviewer’s** **comment**: 3.3 in line 4 what does "p=0.00." means?

**Response**: It shows the degree of heterogeneity among included articles.

**Discussion**

**Reviewer’s** **comment:** The data extraction form is missing

**Response:** The data extraction form is attached as supporting information.

**Reviewer’s** **comment**: lines 9-10 there is no consensus that *LV* defines the clinical form of cutaneous leishmaniasis.

**Response:** We agree that the role of *LRV* in the manifestation of the clinical form of cutaneous leishmaniasis is still debated. As discussed above (introduction comment 2) evidence for and against a defining role of the presence of *LRV* in Leishmania parasites in disease manifestation and severity exist and that more work is required to reach a consensus on this point.

**Reviewer’s** **comment:** lines 13-14 there is no consensus that *LV* causes therapeutic failure.

**Response:** The role of *LRV* on the pathobiology of cutaneous leishmaniasis is still unclear and contradictory evidence on *LRV* positivity and treatment failure have been reported (whereas Valencia *et al*., 2022 showed lack of association, Abtahi et al., 2020, Adaui *et al*., 2016 and Bourreau *et al*., 2016 reported an association). Incorporated in the revised manuscript (page 18, lines 287-289)

**Reviewer’s** **comment:** line 15 separate leishmania from parasite

**Response:** Thank you for pointing out the typing mistake – it has now been corrected in the revised manuscript (page 18, line 290)

**Reviewer’s** **comment**: Figure 4 was not shown in the results

**Response:** It is shown in the discussion section and we add Fig 4 below in the revised manuscript (page 19, line 306)

**Conclusions**

**Reviewer’s** **comment**: Limitations are not clearly discussed and there is no consensus on some of the conclusions

**Response:** We have this now clearly discussed and improved our limitations and conclusions in the revised manuscript (page 21, lines 357-359, 361 and 362).

**Reviewer’s** **comment**: The authors can discuss the article titled "Global status of synchronizing *Leishmania RNA virus* in *Leishmania* parasites: A systematic review with meta‐analysis"…

**Response:** As requested, we discussed this publication in the revised manuscript (page 19, lines 331 and 332, page 20, lines 325-335)

**Figures**

**Reviewer’s** **comment**: The prevalence could be separated by species

**Response**: As advised, we analysed the prevalence of *LRV* in different *Leishmania* species, presented as fig 6

**Conclusions and Limitations**

**Reviewer’s** **comment**: some conclusions are not a consensus in the area

**Response**: This has now been clarified in the revised manuscript (page 21 and 22)

**Reviewer’s** **comment:** It was not discussed that there is little bibliographical information available as a limitation.

**Response:** The limited availability of published data is now pointed out it in the revised manuscript (page 21 and 22)

**Reviewer 3**

**Introduction**

**Reviewer’s** **comment:** In the introduction, some information’s are not based in appropriate references such as case reports and visceral leishmaniasis paper (referring to asymptomatic cases).

**Response:** In the revised manuscript the appropriate references have been added (page 23, references 10, 11)

**Reviewer’s** **comment:** We notice that the authors are not aware that New World CL rarely evolves to self-cure; this information needs correction.

**Response:** This is now clearly stated in the revised manuscript (page 6, lines 110 and 111)

**Reviewer’s** **comment**: Among the articles included some were based in small number of parasites isolates and it is not clear whether in these articles the isolates were randomly included, or they had any bias for the inclusion in the study.

**Response**: We included all articles irrespective of the isolate numbers because the main aim of our study is to show the prevalence of *LRV* in the isolates of different species of *Leishmania* not on the association with clinical manifestations.

**Results**

**Reviewer’s** **comment**: To estimate the prevalence of *LRV*, from the data presented in the Table 2, It seems that the presence of the virus does not relate to clinical presentation and the development of severe forms. There is no other data that show correlation of the presence of the virus and worse disease development. It would be desirable the analysis of the data from articles showing high number of isolates where it would be possible to compare the development of the disease with parasites with and without the virus avoiding the confounding factors such as geographical area and *Leishmania* species diversity.

**Response**: We tried to see if the presence of *LRV* correlates with different clinical manifestations. There are controversial reports on the role of *LRV* in the disease pathology and treatment status. There are not enough published data available to correlate *LRV* presence with disease development and clearly more studies are needed (explained in the revised manuscript pages 21 and 22 Limitation and Conclusion section)

**Conclusions**

**Reviewer’s** **comment**: Conclusions are not fully supported by the presented data. Limitations of the analysis are not clearly described.

**Response**: well taken and we corrected the conclusion and include the limitations in the revised manuscript (pages 21 and 22).

**Reviewer’s** **comment**: The relevance of the study for the public health is not clearly addressed

**Response**: This is now addressed it in the revised manuscript (page 19, lines 311-15, page 21, paragraph 1, lines 10-12)

**Editorial and Data Presentation Modifications**

**Reviewer’s** **comment**: Language revision and edition would be desirable

**Response:** As requested, in the English was corrected by a native English speaker and the edition has been improved.

**Discussion**

**Reviewer’s** **comment:** The correlation of the presence of the virus with the disease development is not clearly presented based on the supporting data.

**Response**: There are not enough published data available to correlate *LRV* presence with disease development and more studies are needed (explained in the revised manuscript pages 21 and 22 limitation and conclusion section)
