# Supplementary material for: Prevalence of Leishmania RNA virus in Leishmania parasites in patients with tegumentary leishmaniasis: A systematic review and meta-analysis
Source: PLoS Negl Trop Dis. 2022 Jun 8;16(6):e0010427. doi: 10.1371/journal.pntd.0010427 (PMC9236231; doi:10.1371/journal.pntd.0010427)
Supplement: Attachment — Submitted filename: point-by point response.docx [file pntd.0010427.s004.docx]

**Response to reviewers’ comments**

We thank the editor for the important comments to improve our manuscript and we address them as follows: The changes and corrections are highlighted in yellow in the revised manuscript.

**Methodology**

**Reviewer’s** **comment:** TOPIC 2.1. Search strategy and eligibility criteria is not clear yet maybe a table for combination word strategy will be necessary, modified in order to make it clearer

**Response:** In response to the reviewer’s comment we have now modified the search strategy and eligibility criteria in the revised manuscript as follows: We first searched the articles using keywords and Medical Subject Headings (MeSH) in the following database: PubMed, Web of Science, Scopus, and Google Scholar; using the following key words:

-	Leishmania RNA virus AND “cutaneous leishmaniasis”;

-	Leishmania RNA virus AND “localized cutaneous leishmaniasis”

-	Leishmania RNA virus AND “mucocutaneous leishmaniasis”;

-	Leishmania RNA virus AND “diffuse leishmaniasis”;

-	Leishmania RNA virus AND “cutaneous leishmaniasis” AND “prevalence”;

-	Leishmania RNA virus AND “localized cutaneous leishmaniasis” AND prevalence”

-	Leishmania RNA virus AND “mucocutaneous leishmaniasis” AND “prevalence”;

-	Leishmania RNA virus AND “diffuse leishmaniasis” AND “prevalence”;

Only peer-reviewed original articles published in English were searched and no restriction was set by publication date, sample size, parasite species, methods used and study settings during article searching. We also had searched from the reference lists of all searched articles to further search similar studies and references.
